# Cardiac Manifestations after Ingestion of a Commercial Desiccant: A Case Report

**DOI:** 10.3390/medicina60010055

**Published:** 2023-12-28

**Authors:** Su-Jeong Shin, Yun-Jeong Kim

**Affiliations:** 1 Department of Emergency Medicine, Yeungnam University Medical Center, Daegu 42415, Republic of Korea; dongle7979@gmail.com; 2 Department of Emergency Medicine, School of Medicine, Kyungpook National University, Daegu 41944, Republic of Korea

**Keywords:** calcium chloride, poisoning, hypercalcemia, cardiomyopathy

## Abstract

*Background and Objectives*: The rise in suicidal attempts has led to an increase in unusual intoxication cases. The ingestion of anhydrous calcium chloride (CaCl_2_) causes direct injury to the gastrointestinal wall via a thermal burn. Therefore, previous reports on CaCl_2_ ingestion primarily considered the gastrointestinal injury. Severe CaCl_2_ intoxication can induce a hypercalcemic crisis, presenting with arrhythmia, acute pancreatitis, and acute kidney injury. This case report details a patient with hematemesis and hypercalcemia following the ingestion of a commercial desiccant. We aimed to report the progression of the case, with a focus on the electrocardiographic manifestations. *Case Presentation*: A 39-year-old female presented at a regional emergency center with blood in her vomit after the ingestion of a commercial desiccant. Bloody emesis was the initial symptom, and various electrolyte imbalances developed during admission. Electrocardiogram (ECG) changes occurred early after hospitalization and disappeared before the electrolyte levels normalized. The patient was maintained in an NPO (Nil Per Os) state throughout her hospital stay. The bloody emesis and abdominal pain resolved quite early, despite her minimal mention of symptoms, possibly due to her suspected negative psychiatric symptoms. *Conclusions*: In this case, we observed dynamic and prolonged multiple electrolyte imbalances along with the early-phase ECG changes, all of which responded well to supportive care. This report adds to the understanding of the diverse manifestations and management of CaCl_2_ intoxication.

## 1. Introduction

Hypercalcemia is defined as a serum calcium level above 2.6 mmol/L (10.7 mg/dL or 5.2 mEq/L). Patients with severe hypercalcemia may exhibit symptoms that include abdominal pain, bone pain, confusion, depression, weakness, kidney stones, or abnormal electrical cardiac rhythms, including cardiac arrest [1]. Calcium is the most abundant extracellular cation in the human body and acts as a secondary messenger and cofactor of several enzymes. In terms of etiology, neoplasia is the most common cause of hypercalcemia in hospitalized and emergency patients, and primary hyperparathyroidism is the predominant cause in outpatient cases [2].

According to the World Health Organization mortality database, over 700,000 people die by committing suicide each year, which makes it the fourth leading cause of death among people under the age of 30 years [3]. A nationwide population study in East Asia revealed that suicide attempts in patients with psychiatric illness occurred more frequently and exhibited a more serious pattern [4]. European data indicated significant gender differences in suicide methods and lethality, with men often choosing more violent and intentional methods [5]. Pesticide intoxication is prevalent in many Asian countries and Latin America, particularly among women, and highlights the significance of self-poisoning, including the use of pesticides or drugs [6].

While chemical intoxication constitutes only a minor portion of poisoning cases, most ingestion-related poisonings are caused by prescription drugs [7]. Deliberate intoxication with chemical substances, excluding certain agricultural products, is rarely reported, and commercial products like dehumidifying agents that contain calcium chloride (CaCl_2_) as their main ingredient are commonly used. Although cases of CaCl_2_ intoxication have been reported, most consist of gastric necrosis with or without surgical treatment and metabolic acidosis that requires continuous renal replacement therapy (CRRT) [8,9,10]. Even though CaCl_2_ is known as a strong stimulant of the skin and mucous membrane, massive CaCl_2_ ingestion accidents sometimes occur, especially in patients with developmental disorders or dementia. We present a case of severe CaCl_2_ intoxication with hematemesis and hypercalcemia and focus on the in-hospital course, with an emphasis on the cardiologic manifestations.

## 2. Case Presentation

A 39-year-old female visited the regional emergency center after vomiting bloody material. She explained that she had ingested a pack of commercial desiccant with a substantial amount of tap water approximately 2 h before her visit, which indicated suicidal intent. Subsequently, she called the rescue center herself, but upon arriving at the emergency department, she was unwilling to answer the medical staff’s questions. Although her eyes remained open throughout, her reactions were extremely limited. The initial examining doctor, from several attempts at cautious history-taking, found no previous specific diseases, including psychiatric conditions. Because of the lack of next of kin or caregivers, obtaining further medical history was challenging. Our focus was primarily on detecting changes in her signs during bedside observation.

**Figure 1:**
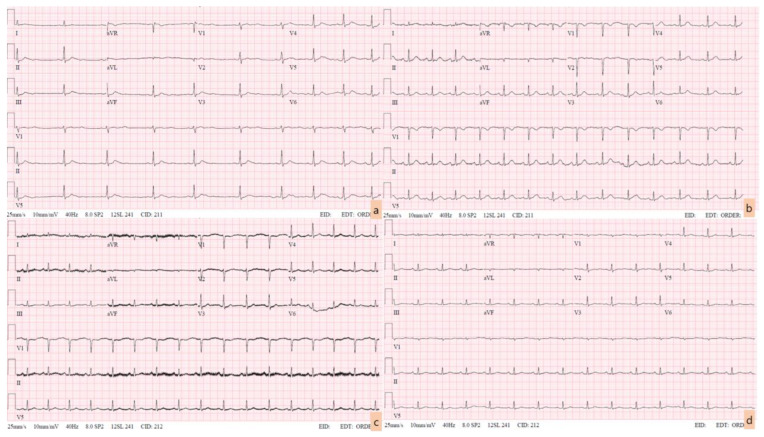
Serial changes in the electrocardiogram (ECG). This is a serial copy of an electrocardiogram taken every day since the first day of hospitalization. The initial ECG showed a relatively short QT interval and subtle changes in the TP segment, including the T wave itself (**a**). Despite a substantial elevation in cardiac enzyme levels, there was no accompanying elevation in the prominent ST-segment (**b**,**c**). The changes in the ECG normalized relatively quickly compared with the electrolyte levels, which had not been corrected for more than a week (**d**).

Her initial vital signs were as follows: blood pressure, 150/90 mmHg; heart rate, 51 beats/min; body temperature, 36.2 °C; respiratory rate, 18 breaths/minute. The electrocardiogram (ECG) showed a relatively short QT interval (QTc 387 ms), a flattened T wave, and a subtle U wave or T-U fusion wave on the II, III, aVF, and precordial leads (Figure 1a). Blood gas analysis revealed metabolic acidosis with respiratory compensation (pH, 7.55; pCO_2_, 20.5 mmHg; pO_2_, 177 mmHg; HCO_3_^−^, 18 mmol/L; SaO_2_, 99.6%) and hyperlactatemia (3.4 mmol/L). Cardiac enzyme levels were all elevated: hsTnI, 0.091 ng/mL (0–0.04); CK-MB, 5.8 ng/mL (0–4.7); myoglobin, 177.5 ng/mL (0–106). A mild electrolyte imbalance was present; the serum potassium was 2.7 mEq/L initially and decreased to 2.5 mEq/L at the 1 h follow-up.

During observation, serial blood sampling was performed. At 12 h post-ingestion, the patient’s ionized calcium level increased to 1.58 mmol/L (1.15–1.29), and her serum calcium and phosphorus levels were 12.3 mg/dL and 19.0 ng/mL, respectively. Her cardiac enzyme levels were also increased: hsTnI was 2.203 ng/mL and CK-MB was 19 ng/mL, but the potassium level normalized to 3.8 mEq/L. At that time, her ECG changed slightly. Even though the heart rate increased to 84 bpm, the corrected QT interval was within the normal range, and the shape of the T wave became more distinguishable. However, there remained a fluttering of the T-P interval in the precordial leads (Figure 1b).

**Figure 2:**
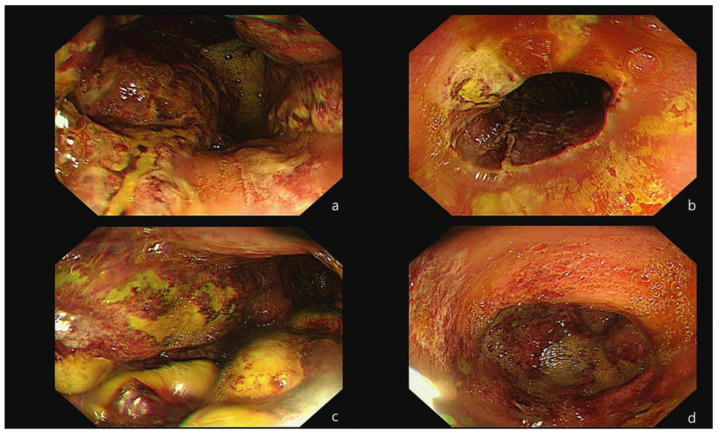
Initial endoscopic test results. The first endoscopic test was performed approximately 20 h post-ingestion. A thick exudate with linear ulceration and hemorrhagic spots were noted in the esophagus (**a**,**b**). Extensive necrosis and multiple ulcerations with brown-grayish discoloration were observed across the whole stomach (**c**,**d**).

Because the bloody vomiting continued occasionally until the next morning, an esophago-gastro-duodenoscopy (EGD) was performed, under consultation, to identify the source of the ongoing bleeding and to assess the severity of the intestinal injury. The procedure revealed a thick exudate with linear ulceration and hemorrhagic spots in the lower esophagus. Additionally, extensive necrosis and multiple ulcerations with brown-grayish discoloration were observed throughout the entire stomach (Figure 2). As a result, the patient was diagnosed with corrosive gastritis, grade IIIB. A decision was made to admit her and to maintain an NPO (Nil Per Os) status until she was re-evaluated for the resumption of a normal diet. Throughout this period, her vital signs remained consistently stable.

On day 3 after admission, there were considerable changes in the patient’s blood test results. Hypocalcemia emerged, which co-existed with mild hypokalemia. The ECG revealed a rather shortened QT interval (Figure 1c), and cardiac enzyme levels decreased. The patient complained of mild epigastric pain but did not report any additional symptoms. Definite tenderness was not prominent on the physical examination. The following day, hypocalcemia, hypophosphatemia, and hypokalemia worsened simultaneously, with persistence of the relatively shortened QT interval on the ECG. Because of the lack of next of kin, financial constraints, and the patient’s reluctance to communicate severe negative symptoms to the medical team, further evaluation, including laboratory tests for vitamins or hormones or a psychiatric interview, could not be conducted.

**Figure 3:**
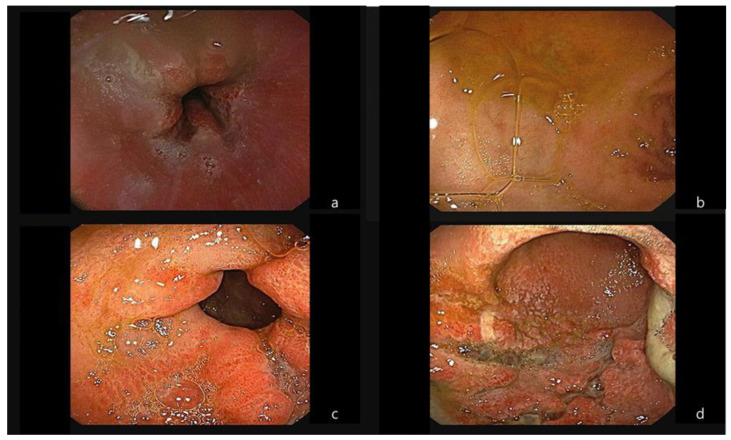
Results of the follow-up endoscopy performed 1 week later. At the gastroesophageal junction level, irregular margins were noted (**a**). Diffuse edema throughout the whole stomach mucosa (**b**,**c**) and multiple focal ulcers were identified that had easy friability and a tendency to bleed (**d**).

For nearly a week, hypocalcemia and hypophosphatemia persisted (Table 1). Meanwhile, the patient complained of hunger, and, although there was the possibility of intestinal lumen injury on a follow-up EGD (Figure 3), a decision was made to start her on a soft diet. There was no aggravation of abdominal pain or signs of gastrointestinal bleeding during this period. However, due to financial constraints regarding medical expenses, the patient had to be transferred.

During hospitalization, the patient was kept on intravenous hydration, not only because she had to keep NPO due to her intestinal injury, but also due to the electrolyte imbalances associated with her transient shock status. On the first day of the visit, the patient’s blood pressure dropped during monitoring, and the bedside echocardiogram performed at the time showed no definite regional wall motion abnormalities. Although the bloody vomitus mixed with repeated vomiting was concerning, the bleeding was not severe enough to cause a significant decrease in hemoglobin levels. As the clinical features were quite unlikely to be accompanied by severe dehydration, her blood pressure was maintained through fluid resuscitation and the use of a small amount of vasoconstrictors. In this case, hypercalcemia was not caused by other specific underlying diseases; rather, it was due to calcium compounds that flowed into the body in large quantities. The patient’s kidney function was well maintained, so separate medication or dialysis was unnecessary, and conservative treatment was considered sufficient.

## 3. Discussion

Most cases of hypercalcemia are caused by primary hyperparathyroidism or malignancy, and hypercalcemia in patients with cancer is associated with a high mortality rate [2]. In a retrospective study, milk–alkali or calcium–alkali syndrome was found to be the third leading cause of hypercalcemia in hospitalized patients without end-stage renal disease [11]. Numerous cases of prescription-drug-induced hypercalcemia have been reported and are typically associated with various comorbidities and renal dysfunction. However, in this case, the patient was relatively healthy physically, with the only presumed serious condition being psychiatric. According to the Society for Endocrinology endocrine emergency guidance, mild hypercalcemia below 3.0 mmol/L is often asymptomatic and does not usually require urgent correction [12]. Correspondingly, in this case, the elevated calcium levels improved with supportive care and without the need for other invasive therapeutic interventions, such as hemodialysis.

Following the initial laboratory test results, a significant augmentation of cardiac enzymes was observed. The follow-up ECGs did not reveal ST-segment or T-wave abnormalities, and definite regional wall-motion restriction was not detected in the bedside echocardiogram. A case report on an elderly patient with ischemic cardiomyopathy and renal insufficiency suggested that hypercalcemia combined with hyperkalemia (with a serum calcium level of 3.55 mmol/L) could induce an elevated ST-segment in the ECG [13]. The authors emphasized that, even in the absence of concerning symptoms and a high probability of myocardial infarction, ECG changes that resemble an ST-segment elevation myocardial infarction should be investigated for potential mimickers. In this case, the serum calcium level was not sufficiently high enough to require aggressive correction, which resulted in the subtle accompanying ECG changes. The most common ECG changes that occur with hypercalcemia are a shortened QT interval and conduction disorders, which are consistent with the aforementioned guidance [2,12,13]. Elevated calcium levels lower the excitation threshold of the myocardium, decrease the cardiac conduction velocity, and shorten the refractory time, especially in phase 2 of the action potential [14].

In addition to the rapid electrolyte imbalance, stress-induced cardiomyopathy (SICMP) can be considered a major cause that explains the rise in cardiac enzyme levels and transient shock status in patients. Burns are one of the major causes of SICMP, along with severe medical conditions, emotional stress, and traumatic events. Left ventricular dysfunction without a definite obstructive coronary lesion, including ST changes in the ECG, can define SICMP [15]. Based on several experimental studies, burn-induced cardiomyopathy may be triggered by mitochondrial damage, which is derived from the surge in catecholamine secretion, the alteration of Ca^2+^ transient proteins, and increased inflammatory responses [16]. Thermal burns induced by CaCl_2_ can cause damage to myocytes, and it can be inferred that calcium absorption greatly contributes to worsening myocyte injury.

In general, the basis for the treatment of hypercalcemia is a massive fluid infusion, and the guidelines recommend 4 to 6 L of 0.9% saline to be intravenously administered within 24 h [12]. If severe renal failure follows or further treatment is required, hemodialysis or intravenous bisphosphonates can be considered. In the present case, the patient was young and had no renal insufficiency, so fluid therapy was enough to treat her condition. During the initial phase of hospital days, the patient showed transient unstable vital signs for a while together with cardiac enzyme elevation, but this was not accompanied by practical cardiac dysfunction based on the bedside echocardiogram. Therefore, a reduction in the intravenous fluid volume was unnecessary.

Due to the patient’s financial constraints, long-term follow-up on her medical condition was not feasible. While the patient’s general condition improved relatively quickly, the electrolyte imbalance persisted. Sudden and extensive elevation of serum calcium levels might suppress the secretion of parathyroid hormone, which leads to bone resorption, a reduction in 1,25-dihydroxyvitamin D_3_, and the subsequent decreased absorption of intestinal calcium [17]. Although these consequences cannot be proven with laboratory results, they can be inferred from the physiological process.

In several case reports about dehumidifier-related poisoning, patients presented with severe intestinal injuries that required surgical emergency intervention, severe metabolic acidosis necessitating CRRT, and even fatal outcomes [8,9,10]. Fortunately, our patient recovered with only supportive care. We assume that the vigorous vomiting immediately after she ingested a large amount of tap water might have limited the CaCl_2_ absorption. This likely contributed to less severe necrosis of the intestinal mucosa and a milder elevation of serum calcium levels. Instead of many tests and expensive treatments, close observation of the patient’s condition and conservative treatment led to a favorable outcome, which can be academically rewarding.

## 4. Conclusions

Sometimes, physicians may encounter patients who have ingested CaCl_2_ in a suicide attempt. Most cases of CaCl_2_ intoxication are related to an upper intestinal injury due to thermal burn, but some of them can experience a severe electrolyte imbalance with cardiomyopathy. Therefore, we have to pay attention to the patients’ cardiac manifestations as well as to their gastrointestinal problems.

## Figures and Tables

**Table 1:** Daily trend of electrolyte levels.

	Reference	Day 1	Day 2	Day 3	Day 4	Day 5	Day 6	Day 7
K	3.5–5.5 mEq/L	3.8	2.9	2.5	3.7			3.5
Ca^2+^	1.15–1.29 mEq/L	1.58	1.14	1.02	1.04	0.99	0.98	1.03
Ca	8.6–10.6 mg/dL	12.3	8.5	7.2	6.9	6.6	6.8	7
P	2.5–4.5 mg/dL	5.6	3.1	1.6	1.1	1.2	1.3	1.2

## Data Availability

The data presented in this study are available on request from the corresponding author. The data are not publicly available due to privacy.
